# 4-Amino-*N*-(4,6-dimethyl­pyrimidin-2-yl)­benzene­sulfonamide–4-nitro­benzoic acid (1/1)

**DOI:** 10.1107/S1600536812019563

**Published:** 2012-05-05

**Authors:** Graham Smith, Urs D. Wermuth

**Affiliations:** a Science and Engineering Faculty, Queensland University of Technology, GPO Box 2434, Brisbane, Queensland 4001, Australia

## Abstract

In the asymmetric unit of the title co-crystal, C_7_H_5_NO_4_·C_12_H_14_N_4_O_2_S, there are two independent but conformationally similar heterodimers, which are formed through inter­molecular N—H⋯O_carb­oxy_ and carbox­yl–pyrimidine O—H⋯N hydrogen-bond pairs, giving a cyclic motif [graph set *R*_2_^2^(8)]. The dihedral angles between the rings in the sulfonamide molecules are 78.77 (8) and 82.33 (9)° while the dihedral angles between the ring and the CO_2_H group in the acids are 2.19 (9) and 7.02 (10)°. A two-dimensional structure parallel to the *ab* plane is generated from the heterodimer units through hydrogen-bonding associations between NH_2_ and sulfone groups. Between neighbouring two-dimensional arrays there are two types of aromatic π–π stacking inter­actions involving either one of the pyrimidine rings and a 4-nitro­benzoic acid mol­ecule [minimum ring centroid separation = 3.5886 (9) Å] or two acid mol­ecules [minimum ring centroid separation = 3.7236 (10) Å].

## Related literature

For background on sulfamethazole as a model for co-crystal formation, see: Caira (2008 ▶). For structures of 1:1 adducts of sulfamethazine with benzoic acid analogues, see: Arman *et al.* (2010 ▶); Caira (1991 ▶, 1992 ▶); Lynch *et al.* (2000 ▶); Patel *et al.* (1988 ▶). For graph-set analysis, see: Etter *et al.* (1990 ▶).
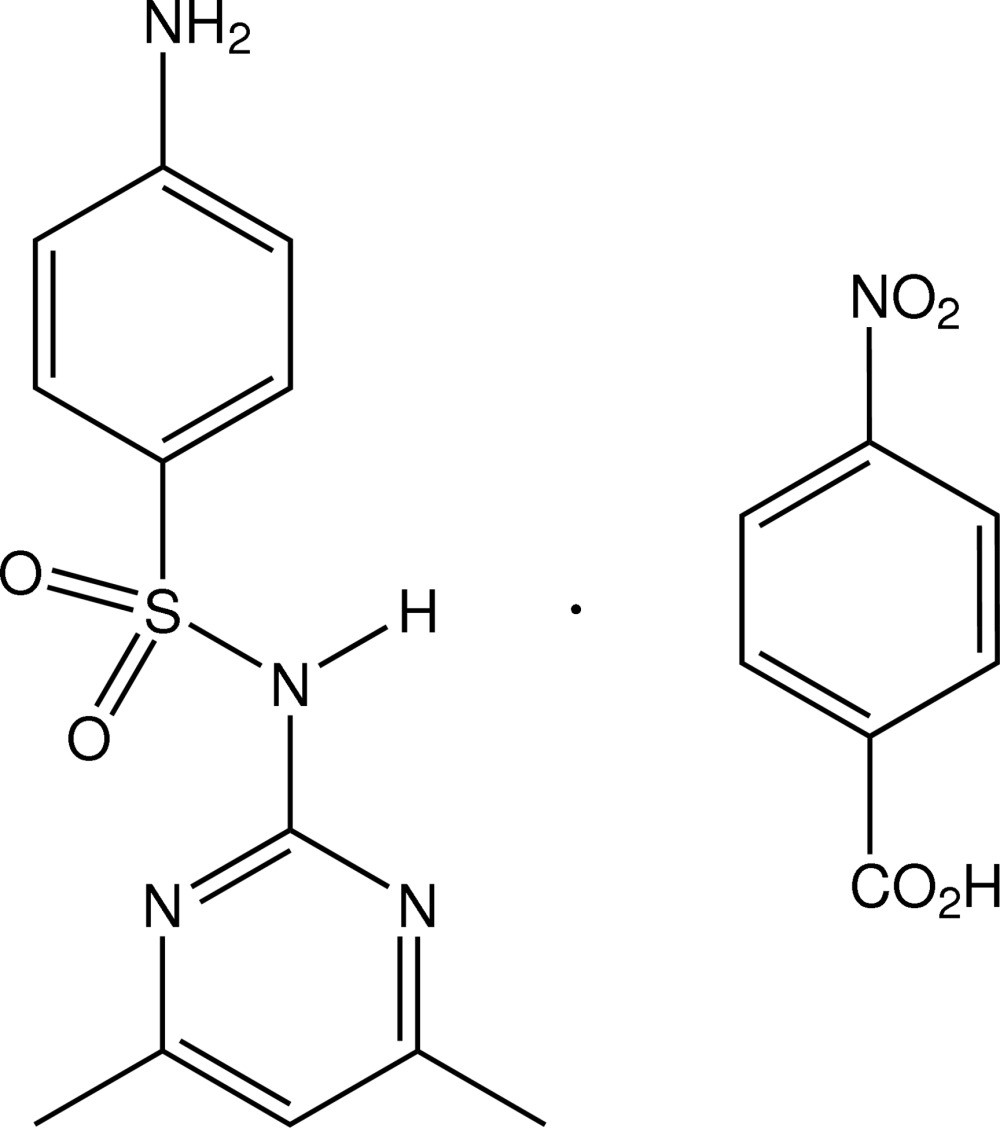


## Experimental

### 

#### Crystal data

C_7_H_5_NO_4_·C_12_H_14_N_4_O_2_S
*M* *_r_* = 445.46
Triclinic, 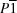

*a* = 8.3483 (3) Å
*b* = 13.8354 (6) Å
*c* = 17.9813 (8) Å
α = 90.810 (4)°
β = 92.841 (4)°
γ = 96.090 (4)°
*V* = 2062.23 (15) Å^3^
*Z* = 4
Mo *K*α radiation
μ = 0.21 mm^−1^
*T* = 200 K
0.35 × 0.35 × 0.30 mm

#### Data collection

Oxford Diffraction Gemini-S CCD detector diffractometer
Absorption correction: multi-scan (*CrysAlis PRO*; Oxford Diffraction, 2010 ▶) *T*_min_ = 0.968, *T*_max_ = 0.988
25486 measured reflections
8077 independent reflections
6075 reflections with *I* > 2σ(*I*)
*R*_int_ = 0.028

#### Refinement

*R*[*F* ^2^ > 2σ(*F* ^2^)] = 0.037
*wR*(*F* ^2^) = 0.093
*S* = 0.99
8077 reflections
595 parameters
H atoms treated by a mixture of independent and constrained refinement
Δρ_max_ = 0.27 e Å^−3^
Δρ_min_ = −0.41 e Å^−3^

### 

Data collection: *CrysAlis PRO* (Oxford Diffraction, 2010 ▶); cell refinement: *CrysAlis PRO*; data reduction: *CrysAlis PRO*; program(s) used to solve structure: *SIR92* (Altomare *et al.*, 1994 ▶); program(s) used to refine structure: *SHELXL97* (Sheldrick, 2008 ▶) in *WinGX* (Farrugia, 1999 ▶); molecular graphics: *PLATON* (Spek, 2009 ▶); software used to prepare material for publication: *PLATON*.

## Supplementary Material

Crystal structure: contains datablock(s) global, I. DOI: 10.1107/S1600536812019563/gk2481sup1.cif

Structure factors: contains datablock(s) I. DOI: 10.1107/S1600536812019563/gk2481Isup2.hkl

Supplementary material file. DOI: 10.1107/S1600536812019563/gk2481Isup3.cml

Additional supplementary materials:  crystallographic information; 3D view; checkCIF report

## Figures and Tables

**Table 1:** Hydrogen-bond geometry (Å, °)

*D*—H⋯*A*	*D*—H	H⋯*A*	*D*⋯*A*	*D*—H⋯*A*
N2*A*—H2*A*⋯O12*C*	0.84 (2)	1.92 (2)	2.758 (2)	178.6 (16)
N2*B*—H2*B*⋯O12*D*	0.88 (2)	1.96 (2)	2.825 (2)	167.9 (17)
N41*A*—H41*A*⋯O12*B*^i^	0.88 (2)	2.23 (2)	3.093 (2)	168.5 (17)
N41*B*—H41*B*⋯O12*A*^ii^	0.86 (2)	2.44 (2)	2.943 (2)	117.9 (15)
N41*A*—H42*A*⋯O11*A*^iii^	0.80 (3)	2.22 (3)	3.002 (2)	164 (2)
N41*B*—H42*B*⋯O11*B*^iii^	0.84 (2)	2.30 (2)	3.066 (2)	152 (2)
O11*C*—H11*C*⋯N1*A*	0.95 (3)	1.74 (3)	2.6829 (18)	175 (2)
O11*D*—H11*D*⋯N3*B*	0.99 (3)	1.67 (3)	2.652 (2)	171 (3)

Symmetry codes: (i) 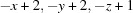; (ii) 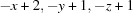; (iii) 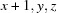.

**Fig. 1.**
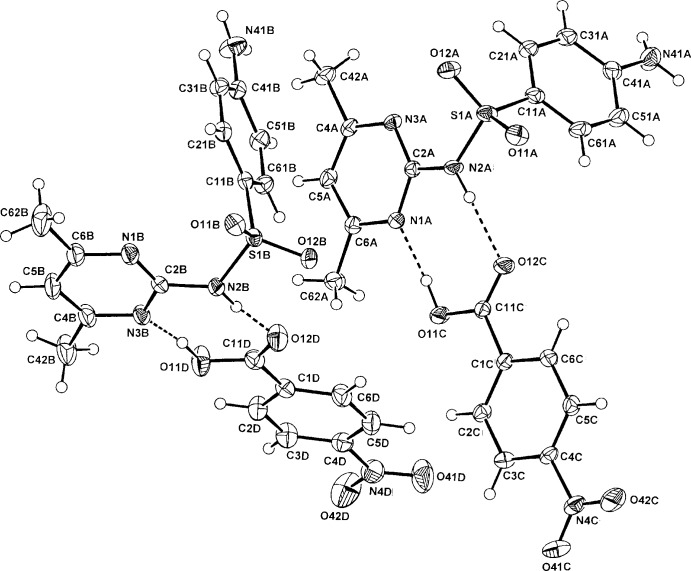
Molecular conformation and atom-numbering scheme for the two independent hydrogen-bonded heteromolecular pairs (A–C and B–D) in the asymmetric unit of the title co-crystal, with inter-species hydrogen bonds shown as dashed lines. Non-hydrogen atoms are shown as 40% probability displacement ellipsoids.

**Fig. 2.**
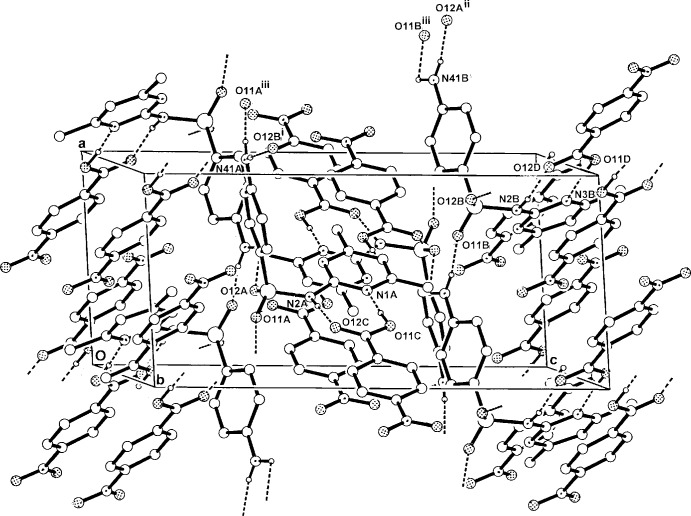
A perspective view of the two-dimensional structure which extends down b, showing hydrogen-bonding associations as dashed lines. For symmetry codes (i)–(iii), see Table 1.

## supplementary crystallographic information

### Comment

The drug sulfamethazine [4-amino-*N*-(4,6-dimethylpyrimidin-2-yl)benzenesulfonamide] has been used as a model for co-crystal formation (Caira, 2008), commonly forming 1:1 adducts with carboxylic acids, particularly the benzoic analogues. The structures of a number of these have been reported, *e.g.* with benzoic acid (Arman *et al.*, 2010); salicylic acid (Patel *et al.*, 1988); anthranilic acid and 4-aminobenzoic acid (Caira, 1991); 4-aminosalicylic acid and acetylsalicylic acid (Caira, 1992) and 2,4-dinitrobenzoic acid (Lynch *et al.*, 2000). In all of these co-crystals, heterodimers are formed through a cyclic hydrogen-bonding motif [graph set *R*^2^_2_(8) (Etter *et al.*, 1990)], involving amine N—H···O_carboxyl_–carboxylic acid O—H···N_pyrimidine_ pairs.

Our 1:1 stoichiometric interaction of sulfamethazine with 4-nitrobenzoic acid also gave a 1:1 co-crystalline adduct C_12_H_14_N_4_O_2_S . C_7_H_5_NO_4_, the title compound, and the structure is reported here. In this co-crystal (Fig. 1), there are two independent molecular pairs (sulfamethazine molecules *A* and *B* with 4-nitrobenzoic acid molecules *C* and *D* respectively), which interact as previously described, giving cyclic *R*^2^_2_(8) hydrogen-bonded heterodimers (Table 1). Intermolecular amine N—H···O_sulfone_ hydrogen-bonding interactions from the heterodimer units (Table 1) generate a two-dimensional structure lying parallel to the *ab* plane (Fig. 2). Between neighbouring two-dimensional structures there are two types of aromatic π–π stacking interactions involving either one of the pyrimidine rings (*A*) and a 4-nitrobenzoic acid molecule *C*^iv^ ([minimum ring centroid separation = 3.5886 (9) Å] or two acid molecules (*D*–*D*^v^) ([minimum ring centroid separation = 3.7236 (10) Å] [symmetry codes: (iv) -*x* + 1, -*y* + 2, -*z* + 1; (v) -*x* + 3, -*y* + 2, -*z* + 2].

There are minor conformational differences between the molecules within the two heterodimer units. The inter-ring dihedral angles between the pyrimidine ring (1) and the benzene ring (2) of the sulfamethazine molecule and the angle between these and the benzene ring of the 4-nitrobenzene group (3) are 78.77 (8), 1.99 (8) and 77.44 (8)°, respectively (the *A*–*C* pair) compared to 82.33 (9), 10.85 (9) and 74.47 (8)° (the *B*–*D* pair).

### Experimental

The title compound was formed in the interaction of 1 mmol quantities of 4-amino-*N*-(4,6-dimethylpyrimidin-2-yl)benzenesulfonamide (sulfamethazine) and 4-nitrobenzoic acid in 50 ml of 50% ethanol–water with 10 min refluxing. Partial evaporation of the solvent gave pale yellow crystal prisms (m.p. 482 K) from which a specimen was cleaved for the X-ray analysis.

### Refinement

Hydrogen atoms potentially involved in hydrogen-bonding interactions were located by difference methods and their positional and isotropic displacement parameters were refined. Other H atoms were included at calculated positions [C—H (aromatic) = 0.93 Å or C—H (methyl) = 0.96 Å] and treated as riding, with *U*_iso_(H) = 1.2*U*_eq_(C) (aromatic) or 1.5*U*_eq_ (C) (methyl).

### Crystal data

**Table:**

C_7_H_5_NO_4_·C_12_H_14_N_4_O_2_S	*Z* = 4
*M**_r_* = 445.46	*F*(000) = 928
Triclinic, *P*1	*D*_x_ = 1.435 Mg m^−^^3^
Hall symbol: -P 1	Melting point: 482 K
*a* = 8.3483 (3) Å	Mo *K*α radiation, λ = 0.71073 Å
*b* = 13.8354 (6) Å	Cell parameters from 10792 reflections
*c* = 17.9813 (8) Å	θ = 3.2–28.7°
α = 90.810 (4)°	µ = 0.21 mm^−^^1^
β = 92.841 (4)°	*T* = 200 K
γ = 96.090 (4)°	Block, pale yellow
*V* = 2062.23 (15) Å^3^	0.35 × 0.35 × 0.30 mm

### Data collection

**Table:**

Oxford Diffraction Gemini-S CCD detector diffractometer	8077 independent reflections
Radiation source: Enhance (Mo) X-ray source	6075 reflections with *I* > 2σ(*I*)
Graphite monochromator	*R*_int_ = 0.028
Detector resolution: 16.0774 pixels mm^-1^	θ_max_ = 26.0°, θ_min_ = 3.2°
ω scans	*h* = −10→10
Absorption correction: multi-scan (*CrysAlis PRO*; Oxford Diffraction, 2010)	*k* = −17→17
*T*_min_ = 0.968, *T*_max_ = 0.988	*l* = −22→22
25486 measured reflections	

### Refinement

**Table:**

Refinement on *F*^2^	Primary atom site location: structure-invariant direct methods
Least-squares matrix: full	Secondary atom site location: difference Fourier map
*R*[*F*^2^ > 2σ(*F*^2^)] = 0.037	Hydrogen site location: inferred from neighbouring sites
*wR*(*F*^2^) = 0.093	H atoms treated by a mixture of independent and constrained refinement
*S* = 0.99	*w* = 1/[σ^2^(*F*_o_^2^) + (0.054*P*)^2^] where *P* = (*F*_o_^2^ + 2*F*_c_^2^)/3
8077 reflections	(Δ/σ)_max_ = 0.002
595 parameters	Δρ_max_ = 0.27 e Å^−^^3^
0 restraints	Δρ_min_ = −0.41 e Å^−^^3^

### Special details

**Table:**

Geometry. Bond distances, angles *etc*. have been calculated using the rounded fractional coordinates. All su's are estimated from the variances of the (full) variance-covariance matrix. The cell e.s.d.'s are taken into account in the estimation of distances, angles and torsion angles
Refinement. Refinement of *F*^2^ against ALL reflections. The weighted *R*-factor *wR* and goodness of fit *S* are based on *F*^2^, conventional *R*-factors *R* are based on *F*, with *F* set to zero for negative *F*^2^. The threshold expression of *F*^2^ > σ(*F*^2^) is used only for calculating *R*-factors(gt) *etc*. and is not relevant to the choice of reflections for refinement. *R*-factors based on *F*^2^ are statistically about twice as large as those based on *F*, and *R*- factors based on ALL data will be even larger.

### Fractional atomic coordinates and isotropic or equivalent isotropic displacement parameters (Å^2^)

**Table:**

	*x*	*y*	*z*	*U*_iso_*/*U*_eq_	
S1A	0.43065 (5)	0.79681 (3)	0.28848 (2)	0.0280 (1)	
O11A	0.31284 (15)	0.85129 (9)	0.25216 (7)	0.0359 (4)	
O12A	0.42294 (15)	0.69500 (9)	0.27420 (7)	0.0354 (4)	
N1A	0.43974 (15)	0.81078 (9)	0.50370 (7)	0.0224 (4)	
N2A	0.40684 (17)	0.81744 (11)	0.37704 (8)	0.0273 (5)	
N3A	0.55850 (16)	0.69806 (9)	0.42680 (7)	0.0237 (4)	
N41A	1.0704 (2)	0.99483 (15)	0.22870 (10)	0.0431 (7)	
C2A	0.47352 (18)	0.77249 (11)	0.43760 (9)	0.0213 (5)	
C4A	0.60735 (19)	0.65360 (12)	0.48885 (9)	0.0248 (5)	
C5A	0.5729 (2)	0.68510 (12)	0.55886 (10)	0.0279 (6)	
C6A	0.49003 (19)	0.76604 (12)	0.56539 (9)	0.0239 (5)	
C11A	0.6208 (2)	0.85296 (13)	0.26962 (9)	0.0277 (6)	
C21A	0.7486 (2)	0.79910 (13)	0.25577 (10)	0.0320 (6)	
C31A	0.8974 (2)	0.84640 (13)	0.24231 (10)	0.0345 (6)	
C41A	0.9242 (2)	0.94797 (13)	0.24233 (9)	0.0306 (6)	
C42A	0.6982 (2)	0.56738 (13)	0.47789 (11)	0.0349 (6)	
C51A	0.7929 (2)	1.00111 (13)	0.25553 (10)	0.0342 (6)	
C61A	0.6457 (2)	0.95425 (13)	0.26920 (10)	0.0319 (6)	
C62A	0.4544 (2)	0.80788 (13)	0.63896 (9)	0.0349 (6)	
S1B	0.82454 (5)	0.68304 (3)	0.76744 (2)	0.0244 (1)	
O11B	0.68139 (14)	0.63072 (9)	0.73454 (6)	0.0316 (4)	
O12B	0.86553 (15)	0.78235 (8)	0.74704 (6)	0.0328 (4)	
N1B	0.70460 (19)	0.53427 (11)	0.88061 (8)	0.0360 (5)	
N2B	0.80838 (18)	0.69373 (11)	0.85831 (8)	0.0265 (5)	
N3B	0.84663 (17)	0.63531 (10)	0.97601 (7)	0.0297 (5)	
N41B	1.3917 (2)	0.47448 (14)	0.72035 (12)	0.0455 (7)	
C2B	0.7850 (2)	0.61641 (12)	0.90656 (9)	0.0257 (5)	
C4B	0.8206 (2)	0.56378 (14)	1.02567 (10)	0.0377 (6)	
C5B	0.7358 (3)	0.47662 (14)	1.00405 (10)	0.0465 (7)	
C6B	0.6801 (3)	0.46280 (14)	0.93033 (10)	0.0425 (7)	
C11B	0.98815 (19)	0.61767 (12)	0.75446 (9)	0.0240 (5)	
C21B	0.9706 (2)	0.52772 (12)	0.71686 (9)	0.0273 (5)	
C31B	1.1044 (2)	0.48082 (13)	0.70492 (9)	0.0306 (6)	
C41B	1.2593 (2)	0.52095 (12)	0.73000 (10)	0.0292 (6)	
C42B	0.8890 (3)	0.58491 (16)	1.10348 (11)	0.0558 (8)	
C51B	1.2744 (2)	0.61104 (13)	0.76910 (10)	0.0313 (6)	
C61B	1.1415 (2)	0.65878 (12)	0.78025 (9)	0.0290 (6)	
C62B	0.5914 (4)	0.36946 (16)	0.90160 (13)	0.0728 (9)	
O11C	0.28879 (15)	0.96896 (9)	0.52899 (6)	0.0318 (4)	
O12C	0.28175 (16)	0.98996 (9)	0.40610 (7)	0.0381 (5)	
O41C	−0.13930 (17)	1.36383 (10)	0.56806 (8)	0.0465 (5)	
O42C	−0.16315 (18)	1.37390 (10)	0.44819 (8)	0.0544 (6)	
N4C	−0.11616 (17)	1.33583 (11)	0.50508 (9)	0.0331 (5)	
C1C	0.14838 (18)	1.09566 (11)	0.48052 (9)	0.0222 (5)	
C2C	0.1110 (2)	1.12500 (12)	0.55148 (9)	0.0259 (5)	
C3C	0.0221 (2)	1.20285 (12)	0.55982 (10)	0.0281 (6)	
C4C	−0.02707 (19)	1.24986 (11)	0.49670 (10)	0.0248 (5)	
C5C	0.0061 (2)	1.22156 (12)	0.42589 (10)	0.0291 (6)	
C6C	0.0943 (2)	1.14346 (12)	0.41817 (9)	0.0279 (6)	
C11C	0.24604 (19)	1.01288 (12)	0.46862 (9)	0.0239 (5)	
O11D	1.05367 (18)	0.78479 (11)	1.02329 (7)	0.0484 (5)	
O12D	1.02623 (17)	0.85230 (10)	0.91233 (7)	0.0466 (5)	
O41D	1.5322 (2)	1.24767 (12)	1.05689 (9)	0.0733 (7)	
O42D	1.5638 (2)	1.17003 (12)	1.15790 (10)	0.0726 (7)	
N4D	1.5070 (2)	1.17635 (12)	1.09470 (10)	0.0439 (6)	
C1D	1.1964 (2)	0.93775 (13)	1.00653 (9)	0.0292 (5)	
C2D	1.2715 (2)	0.93354 (14)	1.07650 (10)	0.0376 (6)	
C3D	1.3738 (2)	1.01145 (14)	1.10513 (11)	0.0399 (7)	
C4D	1.3976 (2)	1.09326 (13)	1.06304 (10)	0.0329 (6)	
C5D	1.3250 (2)	1.09989 (14)	0.99312 (11)	0.0399 (7)	
C6D	1.2240 (2)	1.02123 (14)	0.96483 (10)	0.0374 (6)	
C11D	1.0844 (2)	0.85434 (13)	0.97541 (10)	0.0321 (6)	
H2A	0.370 (2)	0.8702 (15)	0.3853 (11)	0.044 (6)*	
H5A	0.60500	0.65250	0.60100	0.0330*	
H21A	0.73330	0.73150	0.25560	0.0380*	
H31A	0.98210	0.81010	0.23300	0.0410*	
H41A	1.084 (2)	1.0588 (17)	0.2288 (11)	0.048 (6)*	
H42A	1.148 (3)	0.9654 (19)	0.2322 (14)	0.068 (8)*	
H43A	0.78950	0.58540	0.44870	0.0520*	
H44A	0.62880	0.51630	0.45250	0.0520*	
H45A	0.73470	0.54500	0.52550	0.0520*	
H51A	0.80690	1.06870	0.25490	0.0410*	
H61A	0.56060	0.99030	0.27840	0.0380*	
H62A	0.53220	0.86250	0.65170	0.0520*	
H63A	0.46000	0.75940	0.67640	0.0520*	
H64A	0.34820	0.82870	0.63610	0.0520*	
H2B	0.864 (2)	0.7480 (16)	0.8755 (11)	0.046 (6)*	
H5B	0.71600	0.42760	1.03830	0.0560*	
H21B	0.86880	0.49980	0.70000	0.0330*	
H31B	1.09190	0.42120	0.67960	0.0370*	
H41B	1.387 (2)	0.4219 (16)	0.6936 (11)	0.044 (6)*	
H42B	1.483 (3)	0.5016 (16)	0.7337 (12)	0.050 (7)*	
H43B	0.98850	0.62640	1.10210	0.0840*	
H44B	0.90850	0.52510	1.12700	0.0840*	
H45B	0.81370	0.61680	1.13120	0.0840*	
H51B	1.37560	0.63830	0.78750	0.0380*	
H61B	1.15340	0.71870	0.80510	0.0350*	
H62B	0.51600	0.38240	0.86190	0.1090*	
H63B	0.53450	0.33770	0.94110	0.1090*	
H64B	0.66680	0.32820	0.88330	0.1090*	
H2C	0.14570	1.09230	0.59310	0.0310*	
H3C	−0.00410	1.22310	0.60680	0.0340*	
H5C	−0.03000	1.25410	0.38440	0.0350*	
H6C	0.11770	1.12260	0.37090	0.0340*	
H11C	0.343 (3)	0.9142 (19)	0.5171 (14)	0.090 (9)*	
H2D	1.25300	0.87780	1.10440	0.0450*	
H3D	1.42550	1.00870	1.15190	0.0480*	
H5D	1.34360	1.15610	0.96560	0.0480*	
H6D	1.17400	1.02400	0.91760	0.0450*	
H11D	0.976 (3)	0.732 (2)	1.0009 (15)	0.092 (9)*	

### Atomic displacement parameters (Å^2^)

**Table:**

	*U*^11^	*U*^22^	*U*^33^	*U*^12^	*U*^13^	*U*^23^
S1A	0.0325 (2)	0.0323 (3)	0.0213 (2)	0.0143 (2)	−0.0006 (2)	−0.0012 (2)
O11A	0.0363 (7)	0.0457 (8)	0.0284 (7)	0.0191 (6)	−0.0042 (5)	0.0030 (6)
O12A	0.0428 (8)	0.0312 (7)	0.0330 (7)	0.0108 (6)	−0.0010 (6)	−0.0079 (5)
N1A	0.0244 (7)	0.0211 (7)	0.0226 (7)	0.0053 (6)	0.0024 (6)	0.0018 (6)
N2A	0.0360 (9)	0.0266 (8)	0.0226 (8)	0.0177 (7)	0.0035 (6)	0.0007 (6)
N3A	0.0241 (7)	0.0204 (7)	0.0276 (8)	0.0070 (6)	0.0027 (6)	0.0011 (6)
N41A	0.0408 (11)	0.0334 (11)	0.0573 (12)	0.0109 (9)	0.0078 (9)	0.0097 (9)
C2A	0.0200 (8)	0.0201 (8)	0.0242 (9)	0.0034 (7)	0.0024 (7)	0.0018 (7)
C4A	0.0231 (9)	0.0191 (9)	0.0325 (10)	0.0043 (7)	0.0004 (7)	0.0025 (7)
C5A	0.0324 (10)	0.0238 (9)	0.0278 (10)	0.0063 (7)	−0.0029 (7)	0.0050 (7)
C6A	0.0236 (9)	0.0231 (9)	0.0250 (9)	0.0028 (7)	0.0010 (7)	0.0022 (7)
C11A	0.0343 (10)	0.0316 (10)	0.0195 (9)	0.0141 (8)	0.0016 (7)	0.0010 (7)
C21A	0.0397 (11)	0.0271 (10)	0.0321 (10)	0.0146 (8)	0.0062 (8)	0.0029 (8)
C31A	0.0379 (11)	0.0340 (11)	0.0357 (11)	0.0194 (9)	0.0092 (8)	0.0043 (8)
C41A	0.0368 (10)	0.0336 (10)	0.0230 (9)	0.0112 (8)	0.0014 (7)	0.0054 (7)
C42A	0.0393 (11)	0.0279 (10)	0.0400 (11)	0.0154 (8)	0.0021 (8)	0.0021 (8)
C51A	0.0439 (11)	0.0269 (10)	0.0335 (10)	0.0134 (8)	−0.0010 (8)	0.0019 (8)
C61A	0.0370 (11)	0.0324 (11)	0.0292 (10)	0.0193 (8)	−0.0012 (8)	−0.0024 (8)
C62A	0.0471 (12)	0.0354 (11)	0.0243 (10)	0.0146 (9)	0.0015 (8)	0.0018 (8)
S1B	0.0281 (2)	0.0269 (2)	0.0183 (2)	0.0036 (2)	−0.0003 (2)	0.0045 (2)
O11B	0.0270 (6)	0.0409 (7)	0.0263 (7)	0.0030 (5)	−0.0052 (5)	0.0029 (5)
O12B	0.0446 (8)	0.0274 (7)	0.0272 (7)	0.0065 (6)	0.0034 (5)	0.0085 (5)
N1B	0.0519 (10)	0.0304 (9)	0.0240 (8)	−0.0039 (7)	0.0034 (7)	0.0020 (6)
N2B	0.0354 (9)	0.0235 (8)	0.0202 (8)	0.0012 (7)	0.0018 (6)	0.0014 (6)
N3B	0.0377 (9)	0.0301 (8)	0.0204 (8)	−0.0004 (7)	0.0009 (6)	0.0033 (6)
N41B	0.0340 (11)	0.0366 (11)	0.0662 (13)	0.0076 (8)	0.0000 (9)	−0.0114 (9)
C2B	0.0302 (9)	0.0285 (10)	0.0189 (9)	0.0037 (7)	0.0046 (7)	0.0019 (7)
C4B	0.0503 (12)	0.0389 (11)	0.0228 (10)	−0.0025 (9)	0.0036 (8)	0.0064 (8)
C5B	0.0738 (15)	0.0349 (12)	0.0280 (11)	−0.0099 (10)	0.0042 (10)	0.0106 (9)
C6B	0.0675 (14)	0.0310 (11)	0.0266 (10)	−0.0068 (10)	0.0054 (9)	0.0024 (8)
C11B	0.0266 (9)	0.0257 (9)	0.0194 (8)	0.0014 (7)	0.0014 (7)	0.0044 (7)
C21B	0.0305 (9)	0.0277 (10)	0.0218 (9)	−0.0032 (7)	−0.0027 (7)	0.0010 (7)
C31B	0.0366 (10)	0.0253 (10)	0.0291 (10)	0.0013 (8)	0.0003 (8)	−0.0027 (7)
C41B	0.0319 (10)	0.0246 (9)	0.0318 (10)	0.0047 (8)	0.0044 (8)	0.0048 (7)
C42B	0.0830 (17)	0.0518 (14)	0.0270 (11)	−0.0160 (12)	−0.0058 (11)	0.0114 (10)
C51B	0.0272 (9)	0.0291 (10)	0.0363 (10)	−0.0014 (8)	−0.0004 (8)	0.0003 (8)
C61B	0.0316 (10)	0.0238 (9)	0.0304 (10)	−0.0012 (7)	0.0005 (7)	−0.0022 (7)
C62B	0.129 (2)	0.0411 (14)	0.0390 (13)	−0.0308 (14)	−0.0040 (14)	0.0038 (10)
O11C	0.0423 (8)	0.0292 (7)	0.0270 (7)	0.0179 (6)	0.0017 (5)	0.0038 (5)
O12C	0.0565 (9)	0.0342 (8)	0.0290 (7)	0.0256 (6)	0.0106 (6)	0.0056 (6)
O41C	0.0499 (9)	0.0397 (8)	0.0529 (9)	0.0200 (7)	0.0044 (7)	−0.0136 (7)
O42C	0.0679 (10)	0.0431 (9)	0.0584 (10)	0.0341 (8)	0.0029 (8)	0.0120 (7)
N4C	0.0273 (8)	0.0241 (8)	0.0488 (11)	0.0069 (6)	0.0033 (7)	−0.0013 (7)
C1C	0.0211 (8)	0.0189 (8)	0.0269 (9)	0.0029 (7)	0.0019 (7)	0.0018 (7)
C2C	0.0286 (9)	0.0235 (9)	0.0259 (9)	0.0066 (7)	−0.0030 (7)	0.0018 (7)
C3C	0.0305 (10)	0.0267 (10)	0.0274 (10)	0.0054 (7)	0.0014 (7)	−0.0052 (7)
C4C	0.0203 (8)	0.0180 (9)	0.0365 (10)	0.0044 (7)	0.0020 (7)	0.0000 (7)
C5C	0.0307 (10)	0.0277 (10)	0.0305 (10)	0.0085 (8)	0.0033 (7)	0.0090 (7)
C6C	0.0332 (10)	0.0275 (10)	0.0248 (9)	0.0086 (8)	0.0058 (7)	0.0036 (7)
C11C	0.0248 (9)	0.0209 (9)	0.0266 (9)	0.0039 (7)	0.0031 (7)	0.0040 (7)
O11D	0.0623 (10)	0.0428 (9)	0.0337 (8)	−0.0189 (7)	−0.0128 (7)	0.0109 (6)
O12D	0.0654 (10)	0.0410 (8)	0.0280 (8)	−0.0142 (7)	−0.0082 (7)	0.0018 (6)
O41D	0.1005 (14)	0.0516 (10)	0.0588 (11)	−0.0353 (9)	0.0064 (9)	0.0048 (8)
O42D	0.0851 (13)	0.0546 (10)	0.0690 (12)	−0.0144 (9)	−0.0378 (10)	−0.0039 (9)
N4D	0.0429 (10)	0.0384 (10)	0.0480 (11)	−0.0071 (8)	0.0045 (8)	−0.0051 (8)
C1D	0.0290 (9)	0.0332 (10)	0.0252 (9)	0.0020 (8)	0.0026 (7)	−0.0004 (7)
C2D	0.0430 (11)	0.0340 (11)	0.0338 (11)	−0.0041 (9)	−0.0031 (8)	0.0058 (8)
C3D	0.0412 (11)	0.0428 (12)	0.0330 (11)	−0.0037 (9)	−0.0080 (8)	0.0026 (9)
C4D	0.0291 (10)	0.0327 (11)	0.0360 (11)	−0.0012 (8)	0.0033 (8)	−0.0054 (8)
C5D	0.0473 (12)	0.0328 (11)	0.0383 (11)	−0.0039 (9)	0.0054 (9)	0.0064 (9)
C6D	0.0475 (12)	0.0378 (11)	0.0256 (10)	−0.0009 (9)	−0.0010 (8)	0.0022 (8)
C11D	0.0365 (10)	0.0334 (11)	0.0263 (10)	0.0024 (8)	0.0022 (8)	0.0005 (8)

### Geometric parameters (Å, º)

**Table:**

S1A—O11A	1.4386 (13)	C42A—H44A	0.9600
S1A—O12A	1.4224 (13)	C42A—H43A	0.9600
S1A—N2A	1.6393 (15)	C42A—H45A	0.9600
S1A—C11A	1.7429 (18)	C51A—H51A	0.9300
S1B—O12B	1.4375 (12)	C61A—H61A	0.9300
S1B—N2B	1.6520 (15)	C62A—H64A	0.9600
S1B—C11B	1.7395 (17)	C62A—H62A	0.9600
S1B—O11B	1.4270 (12)	C62A—H63A	0.9600
O11C—C11C	1.304 (2)	C4B—C5B	1.373 (3)
O12C—C11C	1.223 (2)	C4B—C42B	1.498 (3)
O41C—N4C	1.223 (2)	C5B—C6B	1.387 (3)
O42C—N4C	1.224 (2)	C6B—C62B	1.491 (3)
O11C—H11C	0.95 (3)	C11B—C21B	1.398 (2)
O11D—C11D	1.314 (2)	C11B—C61B	1.399 (2)
O12D—C11D	1.210 (2)	C21B—C31B	1.374 (2)
O41D—N4D	1.212 (2)	C31B—C41B	1.402 (2)
O42D—N4D	1.217 (3)	C41B—C51B	1.413 (2)
O11D—H11D	0.99 (3)	C51B—C61B	1.372 (2)
N1A—C6A	1.348 (2)	C5B—H5B	0.9300
N1A—C2A	1.349 (2)	C21B—H21B	0.9300
N2A—C2A	1.384 (2)	C31B—H31B	0.9300
N3A—C2A	1.328 (2)	C42B—H45B	0.9600
N3A—C4A	1.348 (2)	C42B—H43B	0.9600
N41A—C41A	1.355 (2)	C42B—H44B	0.9600
N2A—H2A	0.84 (2)	C51B—H51B	0.9300
N41A—H41A	0.88 (2)	C61B—H61B	0.9300
N41A—H42A	0.80 (3)	C62B—H64B	0.9600
N1B—C2B	1.324 (2)	C62B—H63B	0.9600
N1B—C6B	1.349 (2)	C62B—H62B	0.9600
N2B—C2B	1.391 (2)	C1C—C11C	1.494 (2)
N3B—C2B	1.338 (2)	C1C—C6C	1.390 (2)
N3B—C4B	1.350 (2)	C1C—C2C	1.394 (2)
N41B—C41B	1.354 (2)	C2C—C3C	1.382 (2)
N2B—H2B	0.88 (2)	C3C—C4C	1.382 (2)
N41B—H41B	0.86 (2)	C4C—C5C	1.377 (3)
N41B—H42B	0.84 (2)	C5C—C6C	1.380 (2)
N4C—C4C	1.478 (2)	C2C—H2C	0.9300
N4D—C4D	1.479 (2)	C3C—H3C	0.9300
C4A—C42A	1.496 (2)	C5C—H5C	0.9300
C4A—C5A	1.380 (2)	C6C—H6C	0.9300
C5A—C6A	1.384 (2)	C1D—C6D	1.391 (3)
C6A—C62A	1.493 (2)	C1D—C11D	1.490 (2)
C11A—C21A	1.395 (2)	C1D—C2D	1.382 (2)
C11A—C61A	1.395 (3)	C2D—C3D	1.379 (3)
C21A—C31A	1.375 (2)	C3D—C4D	1.373 (3)
C31A—C41A	1.399 (3)	C4D—C5D	1.377 (3)
C41A—C51A	1.411 (2)	C5D—C6D	1.379 (3)
C51A—C61A	1.363 (2)	C2D—H2D	0.9300
C5A—H5A	0.9300	C3D—H3D	0.9300
C21A—H21A	0.9300	C5D—H5D	0.9300
C31A—H31A	0.9300	C6D—H6D	0.9300
			
O11A—S1A—O12A	119.17 (8)	C5B—C4B—C42B	123.01 (17)
O11A—S1A—N2A	102.91 (8)	N3B—C4B—C5B	120.29 (16)
O11A—S1A—C11A	107.51 (8)	N3B—C4B—C42B	116.70 (16)
O12A—S1A—N2A	109.82 (8)	C4B—C5B—C6B	118.97 (17)
O12A—S1A—C11A	110.09 (8)	C5B—C6B—C62B	122.51 (18)
N2A—S1A—C11A	106.48 (8)	N1B—C6B—C5B	120.99 (18)
O12B—S1B—N2B	101.89 (7)	N1B—C6B—C62B	116.51 (17)
O12B—S1B—C11B	109.32 (8)	C21B—C11B—C61B	119.77 (15)
N2B—S1B—C11B	106.68 (8)	S1B—C11B—C21B	121.78 (13)
O11B—S1B—C11B	109.61 (8)	S1B—C11B—C61B	118.41 (13)
O11B—S1B—O12B	118.86 (7)	C11B—C21B—C31B	119.71 (15)
O11B—S1B—N2B	109.66 (7)	C21B—C31B—C41B	121.44 (16)
C11C—O11C—H11C	110.7 (15)	N41B—C41B—C31B	122.12 (16)
C11D—O11D—H11D	110.8 (16)	C31B—C41B—C51B	118.07 (15)
C2A—N1A—C6A	116.89 (13)	N41B—C41B—C51B	119.77 (16)
S1A—N2A—C2A	127.86 (12)	C41B—C51B—C61B	120.70 (16)
C2A—N3A—C4A	115.69 (13)	C11B—C61B—C51B	120.29 (15)
C2A—N2A—H2A	117.0 (13)	C4B—C5B—H5B	121.00
S1A—N2A—H2A	113.2 (14)	C6B—C5B—H5B	120.00
H41A—N41A—H42A	119 (2)	C11B—C21B—H21B	120.00
C41A—N41A—H42A	118.7 (19)	C31B—C21B—H21B	120.00
C41A—N41A—H41A	119.9 (11)	C41B—C31B—H31B	119.00
C2B—N1B—C6B	115.97 (15)	C21B—C31B—H31B	119.00
S1B—N2B—C2B	125.01 (12)	H43B—C42B—H45B	109.00
C2B—N3B—C4B	116.62 (14)	C4B—C42B—H43B	109.00
S1B—N2B—H2B	110.2 (13)	H44B—C42B—H45B	109.00
C2B—N2B—H2B	118.1 (13)	C4B—C42B—H45B	109.00
H41B—N41B—H42B	117.6 (19)	H43B—C42B—H44B	110.00
C41B—N41B—H41B	121.5 (11)	C4B—C42B—H44B	109.00
C41B—N41B—H42B	119.9 (16)	C41B—C51B—H51B	120.00
O41C—N4C—C4C	118.18 (15)	C61B—C51B—H51B	120.00
O41C—N4C—O42C	124.27 (15)	C11B—C61B—H61B	120.00
O42C—N4C—C4C	117.55 (15)	C51B—C61B—H61B	120.00
O41D—N4D—O42D	123.73 (18)	H62B—C62B—H64B	109.00
O42D—N4D—C4D	118.08 (16)	H63B—C62B—H64B	109.00
O41D—N4D—C4D	118.17 (17)	C6B—C62B—H63B	109.00
N1A—C2A—N2A	113.44 (13)	C6B—C62B—H64B	109.00
N1A—C2A—N3A	126.78 (14)	H62B—C62B—H63B	110.00
N2A—C2A—N3A	119.77 (14)	C6B—C62B—H62B	109.00
C5A—C4A—C42A	121.72 (15)	C2C—C1C—C6C	120.19 (14)
N3A—C4A—C5A	121.65 (15)	C6C—C1C—C11C	117.96 (14)
N3A—C4A—C42A	116.62 (15)	C2C—C1C—C11C	121.85 (14)
C4A—C5A—C6A	118.98 (16)	C1C—C2C—C3C	119.79 (15)
N1A—C6A—C62A	117.53 (14)	C2C—C3C—C4C	118.46 (16)
C5A—C6A—C62A	122.59 (15)	N4C—C4C—C3C	118.98 (15)
N1A—C6A—C5A	119.88 (15)	N4C—C4C—C5C	118.03 (15)
C21A—C11A—C61A	119.40 (16)	C3C—C4C—C5C	122.97 (15)
S1A—C11A—C61A	119.01 (13)	C4C—C5C—C6C	118.09 (16)
S1A—C11A—C21A	121.59 (14)	C1C—C6C—C5C	120.48 (15)
C11A—C21A—C31A	119.68 (16)	O11C—C11C—C1C	115.10 (14)
C21A—C31A—C41A	121.45 (16)	O12C—C11C—C1C	120.96 (15)
C31A—C41A—C51A	118.00 (16)	O11C—C11C—O12C	123.95 (15)
N41A—C41A—C31A	121.62 (17)	C1C—C2C—H2C	120.00
N41A—C41A—C51A	120.37 (17)	C3C—C2C—H2C	120.00
C41A—C51A—C61A	120.54 (16)	C2C—C3C—H3C	121.00
C11A—C61A—C51A	120.93 (16)	C4C—C3C—H3C	121.00
C6A—C5A—H5A	121.00	C4C—C5C—H5C	121.00
C4A—C5A—H5A	121.00	C6C—C5C—H5C	121.00
C11A—C21A—H21A	120.00	C1C—C6C—H6C	120.00
C31A—C21A—H21A	120.00	C5C—C6C—H6C	120.00
C21A—C31A—H31A	119.00	C2D—C1D—C11D	120.85 (16)
C41A—C31A—H31A	119.00	C6D—C1D—C11D	119.40 (15)
C4A—C42A—H44A	109.00	C2D—C1D—C6D	119.74 (16)
C4A—C42A—H45A	109.00	C1D—C2D—C3D	120.44 (17)
C4A—C42A—H43A	110.00	C2D—C3D—C4D	118.56 (17)
H43A—C42A—H45A	109.00	N4D—C4D—C3D	117.89 (16)
H43A—C42A—H44A	109.00	C3D—C4D—C5D	122.53 (17)
H44A—C42A—H45A	109.00	N4D—C4D—C5D	119.58 (16)
C41A—C51A—H51A	120.00	C4D—C5D—C6D	118.38 (17)
C61A—C51A—H51A	120.00	C1D—C6D—C5D	120.34 (17)
C11A—C61A—H61A	120.00	O11D—C11D—C1D	113.51 (15)
C51A—C61A—H61A	120.00	O12D—C11D—C1D	122.71 (16)
H62A—C62A—H63A	109.00	O11D—C11D—O12D	123.78 (17)
H63A—C62A—H64A	109.00	C1D—C2D—H2D	120.00
H62A—C62A—H64A	109.00	C3D—C2D—H2D	120.00
C6A—C62A—H62A	109.00	C2D—C3D—H3D	121.00
C6A—C62A—H64A	110.00	C4D—C3D—H3D	121.00
C6A—C62A—H63A	109.00	C4D—C5D—H5D	121.00
N1B—C2B—N2B	118.33 (15)	C6D—C5D—H5D	121.00
N1B—C2B—N3B	127.13 (15)	C1D—C6D—H6D	120.00
N2B—C2B—N3B	114.53 (14)	C5D—C6D—H6D	120.00
			
O11A—S1A—N2A—C2A	170.32 (14)	S1A—C11A—C61A—C51A	179.27 (14)
O12A—S1A—N2A—C2A	42.43 (17)	C61A—C11A—C21A—C31A	0.4 (3)
C11A—S1A—N2A—C2A	−76.75 (16)	C21A—C11A—C61A—C51A	−0.1 (3)
O11A—S1A—C11A—C21A	−141.58 (14)	C11A—C21A—C31A—C41A	0.2 (3)
O11A—S1A—C11A—C61A	39.11 (16)	C21A—C31A—C41A—C51A	−0.9 (3)
O12A—S1A—C11A—C21A	−10.30 (17)	C21A—C31A—C41A—N41A	−179.64 (17)
O12A—S1A—C11A—C61A	170.39 (13)	C31A—C41A—C51A—C61A	1.2 (3)
N2A—S1A—C11A—C21A	108.69 (15)	N41A—C41A—C51A—C61A	179.95 (17)
N2A—S1A—C11A—C61A	−70.62 (15)	C41A—C51A—C61A—C11A	−0.8 (3)
N2B—S1B—C11B—C21B	118.51 (14)	N3B—C4B—C5B—C6B	1.1 (3)
N2B—S1B—C11B—C61B	−63.86 (15)	C42B—C4B—C5B—C6B	−178.5 (2)
O11B—S1B—N2B—C2B	59.69 (16)	C4B—C5B—C6B—N1B	−1.7 (3)
O12B—S1B—N2B—C2B	−173.51 (14)	C4B—C5B—C6B—C62B	178.1 (2)
C11B—S1B—N2B—C2B	−58.93 (16)	S1B—C11B—C61B—C51B	−177.98 (13)
O11B—S1B—C11B—C21B	−0.14 (16)	S1B—C11B—C21B—C31B	176.99 (13)
O11B—S1B—C11B—C61B	177.48 (12)	C21B—C11B—C61B—C51B	−0.3 (2)
O12B—S1B—C11B—C21B	−132.04 (14)	C61B—C11B—C21B—C31B	−0.6 (2)
O12B—S1B—C11B—C61B	45.59 (15)	C11B—C21B—C31B—C41B	0.3 (3)
C2A—N1A—C6A—C5A	0.4 (2)	C21B—C31B—C41B—N41B	178.27 (18)
C2A—N1A—C6A—C62A	179.66 (14)	C21B—C31B—C41B—C51B	0.8 (3)
C6A—N1A—C2A—N3A	−3.7 (2)	C31B—C41B—C51B—C61B	−1.8 (3)
C6A—N1A—C2A—N2A	175.03 (14)	N41B—C41B—C51B—C61B	−179.24 (18)
S1A—N2A—C2A—N3A	−5.7 (2)	C41B—C51B—C61B—C11B	1.5 (3)
S1A—N2A—C2A—N1A	175.49 (12)	C11C—C1C—C6C—C5C	−178.81 (15)
C2A—N3A—C4A—C5A	−1.1 (2)	C2C—C1C—C11C—O12C	−177.81 (16)
C4A—N3A—C2A—N1A	4.0 (2)	C6C—C1C—C11C—O11C	−177.86 (14)
C4A—N3A—C2A—N2A	−174.64 (14)	C2C—C1C—C11C—O11C	2.0 (2)
C2A—N3A—C4A—C42A	177.89 (14)	C6C—C1C—C2C—C3C	−1.0 (2)
C6B—N1B—C2B—N3B	1.3 (3)	C11C—C1C—C2C—C3C	179.13 (15)
C2B—N1B—C6B—C5B	0.5 (3)	C2C—C1C—C6C—C5C	1.4 (2)
C6B—N1B—C2B—N2B	−177.64 (17)	C6C—C1C—C11C—O12C	2.4 (2)
C2B—N1B—C6B—C62B	−179.3 (2)	C1C—C2C—C3C—C4C	−0.2 (2)
S1B—N2B—C2B—N1B	−30.2 (2)	C2C—C3C—C4C—N4C	−177.39 (15)
S1B—N2B—C2B—N3B	150.71 (13)	C2C—C3C—C4C—C5C	1.2 (3)
C2B—N3B—C4B—C5B	0.5 (3)	C3C—C4C—C5C—C6C	−0.9 (3)
C2B—N3B—C4B—C42B	−179.80 (17)	N4C—C4C—C5C—C6C	177.70 (15)
C4B—N3B—C2B—N2B	177.15 (15)	C4C—C5C—C6C—C1C	−0.4 (2)
C4B—N3B—C2B—N1B	−1.9 (3)	C6D—C1D—C2D—C3D	−0.1 (3)
O41C—N4C—C4C—C5C	−176.68 (15)	C11D—C1D—C2D—C3D	−179.39 (16)
O41C—N4C—C4C—C3C	2.0 (2)	C2D—C1D—C6D—C5D	−0.4 (3)
O42C—N4C—C4C—C3C	−177.47 (15)	C11D—C1D—C6D—C5D	178.87 (16)
O42C—N4C—C4C—C5C	3.9 (2)	C2D—C1D—C11D—O11D	6.9 (2)
O42D—N4D—C4D—C5D	176.81 (17)	C2D—C1D—C11D—O12D	−174.15 (17)
O42D—N4D—C4D—C3D	−3.7 (3)	C6D—C1D—C11D—O11D	−172.39 (16)
O41D—N4D—C4D—C3D	177.85 (17)	C6D—C1D—C11D—O12D	6.6 (3)
O41D—N4D—C4D—C5D	−1.6 (3)	C1D—C2D—C3D—C4D	0.8 (3)
N3A—C4A—C5A—C6A	−1.8 (2)	C2D—C3D—C4D—N4D	179.69 (16)
C42A—C4A—C5A—C6A	179.26 (15)	C2D—C3D—C4D—C5D	−0.9 (3)
C4A—C5A—C6A—N1A	2.2 (2)	N4D—C4D—C5D—C6D	179.77 (16)
C4A—C5A—C6A—C62A	−177.08 (15)	C3D—C4D—C5D—C6D	0.4 (3)
S1A—C11A—C21A—C31A	−178.96 (14)	C4D—C5D—C6D—C1D	0.3 (3)

### Hydrogen-bond geometry (Å, º)

**Table:**

*D*—H···*A*	*D*—H	H···*A*	*D*···*A*	*D*—H···*A*
N2*A*—H2*A*···O12*C*	0.84 (2)	1.92 (2)	2.758 (2)	178.6 (16)
N2*B*—H2*B*···O12*D*	0.88 (2)	1.96 (2)	2.825 (2)	167.9 (17)
N41*A*—H41*A*···O12*B*^i^	0.88 (2)	2.23 (2)	3.093 (2)	168.5 (17)
N41*B*—H41*B*···O12*A*^ii^	0.86 (2)	2.44 (2)	2.943 (2)	117.9 (15)
N41*A*—H42*A*···O11*A*^iii^	0.80 (3)	2.22 (3)	3.002 (2)	164 (2)
N41*B*—H42*B*···O11*B*^iii^	0.84 (2)	2.30 (2)	3.066 (2)	152 (2)
O11*C*—H11*C*···N1*A*	0.95 (3)	1.74 (3)	2.6829 (18)	175 (2)
O11*D*—H11*D*···N3*B*	0.99 (3)	1.67 (3)	2.652 (2)	171 (3)
C2*D*—H2*D*···O11*D*	0.93	2.41	2.727 (2)	100
C5*A*—H5*A*···O11*B*	0.93	2.48	3.361 (2)	157
C6*C*—H6*C*···O12*B*^iv^	0.93	2.51	3.179 (2)	129
C62*B*—H63*B*···O41*D*^v^	0.96	2.44	3.313 (3)	151

Symmetry codes: (i) −*x*+2, −*y*+2, −*z*+1; (ii) −*x*+2, −*y*+1, −*z*+1; (iii) *x*+1, *y*, *z*; (iv) −*x*+1, −*y*+2, −*z*+1; (v) *x*−1, *y*−1, *z*.
